# Material Properties from Air Puff Corneal Deformation by Numerical Simulations on Model Corneas

**DOI:** 10.1371/journal.pone.0165669

**Published:** 2016-10-28

**Authors:** Nandor Bekesi, Carlos Dorronsoro, Andrés de la Hoz, Susana Marcos

**Affiliations:** Instituto de Óptica “Daza de Valdés”, Consejo Superior de Investigaciones Científicas, Madrid, Spain; Oklahoma State University Center for Health Sciences, UNITED STATES

## Abstract

**Objective:**

To validate a new method for reconstructing corneal biomechanical properties from air puff corneal deformation images using hydrogel polymer model corneas and porcine corneas.

**Methods:**

Air puff deformation imaging was performed on model eyes with artificial corneas made out of three different hydrogel materials with three different thicknesses and on porcine eyes, at constant intraocular pressure of 15 mmHg. The cornea air puff deformation was modeled using finite elements, and hyperelastic material parameters were determined through inverse modeling, minimizing the difference between the simulated and the measured central deformation amplitude and central-peripheral deformation ratio parameters. Uniaxial tensile tests were performed on the model cornea materials as well as on corneal strips, and the results were compared to stress-strain simulations assuming the reconstructed material parameters.

**Results:**

The measured and simulated spatial and temporal profiles of the air puff deformation tests were in good agreement (< 7% average discrepancy). The simulated stress-strain curves of the studied hydrogel corneal materials fitted well the experimental stress-strain curves from uniaxial extensiometry, particularly in the 0–0.4 range. Equivalent Young´s moduli of the reconstructed material properties from air-puff were 0.31, 0.58 and 0.48 MPa for the three polymer materials respectively which differed < 1% from those obtained from extensiometry. The simulations of the same material but different thickness resulted in similar reconstructed material properties. The air-puff reconstructed average equivalent Young´s modulus of the porcine corneas was 1.3 MPa, within 18% of that obtained from extensiometry.

**Conclusions:**

Air puff corneal deformation imaging with inverse finite element modeling can retrieve material properties of model hydrogel polymer corneas and real corneas, which are in good correspondence with those obtained from uniaxial extensiometry, suggesting that this is a promising technique to retrieve quantitative corneal biomechanical properties.

## Introduction

Measuring mechanical properties of soft biological tissues is not only important to monitor progression in diseases that alter biomechanical properties and cause mechanical symptoms (e.g. limiting mobility of joints), but also as early diagnostic biomarkers. For example, tumor progression has been shown to entail biomechanical changes in tissue [1] and chronic kidney disease is associated with arterial stiffening [2]. Measurements of mechanical properties of tissue are also instrumental in assessing treatments and in monitoring healing processes [3].

In ophthalmology, measurement of the mechanical properties of corneal tissue is particularly well suited in diagnostics of corneal disease and in screening patients for corneal surgery. Keratoconus is a disease of the cornea, which causes pathological weakening of the corneal stroma, resulting in changing its shape and degrading vision radically [4]. It is well accepted that the biomechanical properties of the cornea in keratoconus differ significantly from those in normal corneas [5]. In refractive surgery, where corneal thickness is reduced as a result of corneal reshaping to eliminate refractive errors, pre-operative measurements of corneal biomechanics could help identifying patients at risk of iatrogenic ectasia [6]. Individual knowledge of the cornea can help in customizing certain treatments for keratoconus such as intracorneal ring segment implants [7] or to monitor the effectiveness of corneal collagen cross-linking [8].

Most of the corneal biomechanical properties data reported in the literature were obtained on ex vivo samples, using tensile tests [9] or inflation [10]. A disadvantage of these tests is that post-mortem time influences the hydration state of the cornea, which in turn affects the mechanical properties [11]. Also, tests requiring the use of corneal strips are affected by the orientation and density of the collagen fibers [12–14], and changes in the boundary conditions [15].

Recently some methods measuring corneal biomechanical properties in vivo have been demonstrated, including cantilever indentation [16], ultrasound [17] and magnetic resonance elastography [18]. These techniques typically require direct contact of a probe with the patient’s cornea. There is a demand for a less invasive, non-contact method. Brillouin spectroscopy [19] and Optical Coherence Tomography (OCT)-based elastography [20] are fully non-invasive and have been used to compare changes in corneal parameters with treatment (e.g. untreated vs. cross-linked corneas). However, they generally do not provide absolute inherent mechanical parameters but associated parameters (i.e. Brillouin modulus) and therefore they are primarily used to account for relative changes (i.e. with cross-linking). A non-invasive in vivo characterization method is needed, which provides corneal biomechanical properties that can be used as biomarkers for keratoconus, and absolute patient-specific mechanical parameters that can be introduced in mechanical calculations and simulations, therefore allowing more predictable treatments.

Air puff corneal deformation is frequently used in ophthalmology, more generally to measure the intraocular pressure (IOP) by the so-called applanation tonometry. Air puff corneal deformation in general utilizes a quick air pulse to deform the cornea non-invasively, unlike Goldmann or Perkins tonometry, which use an intender that touches the cornea. Measurements of IOP are in turn affected by individual variations of corneal mechanical properties. Air puff corneal deformation involves the use of a rapid air pulse deforming the cornea, and the deformation event is captured by an adequately fast imaging system e.g. OCT [21, 22] or Scheimpflug [23], such as the commercial instrument Corvis ST (Oculus, Germany). Kling et al. in [23] studied the contributing factors to air puff corneal deformation and found that IOP, elastic and viscoelastic parameters and also their distribution along the depth of the corneal stroma have effect on the amplitude and shape of corneal deformation.

Corneal deformation parameters also depend on the characteristics of the air pulse: its maximum pressure, spatial distribution and temporal profile, which may vary in different devices (typically 20 ms long, but even down to 1 μs [24]). While corneal deformation parameters (i.e. corneal deformation amplitude) obtained from these instruments are indirectly related with corneal biomechanics, the inherent corneal material parameters (i.e. Young’s modulus, viscoelastic parameters) are better metrics for diagnostics and treatment planning and monitoring. In particular, finite element (FE) analysis is a standard numerical tool to study mechanical behavior of structures, even soft materials [25], including ocular tissues [26–28]. Using temporal and spatial corneal deformation data as input, optimization algorithms allows in principle retrieving corneal biomechanical parameters. In an earlier study, we reported a 2-step optimization procedure to retrieve corneal mechanical properties from corneal deformation data [8]. Simonini et al. studied the corneal air puff deformation analytically and numerically [29]. Other authors also used FE inverse modeling to obtain hyperelastic [30] and visco-elastic properties of the cornea [10]. However, to our knowledge, a validation of the approach has not been reported. In this study, we used corneal phantoms as well as real corneas to demonstrate the feasibility of the reconstruction of material properties from air puff corneal deformation data.

The use of phantoms is a common way to model corneal biomechanics. The Corvis ST device is also calibrated by rubber corneal phantoms. Li et al. studied corneal viscoelasticity in gelatin phantom and mouse cornea [24]. Liu et al. in [31] measured ultrasound wave propagation in soft contact lenses. Correia et al. [32] measured Corvis ST corneal deformation amplitude on artificial corneas lathed on contact lens materials.

In this study we demonstrate an optimization technique for obtaining material properties non-invasively combining air puff deformation imaging on hydrogel phantoms and inverse FE modeling. The resulting mechanical properties of the model corneas (MC) were compared to standard material test results. The mechanical parameters from the proposed method showed good agreement with the standard tensile measurements, regardless of the thickness of the cornea. We further demonstrated the reconstruction as well as the correspondence between material properties retrieved from air puff corneal deformation and uniaxial extensiometry in porcine eyes. This study represents a validation of the method for applications in cornea.

## Methods

Air puff corneal deformation imaging was carried out using a Scheimpflug-based system on model and porcine corneas. The air puff test configuration was modeled numerically using FE modeling. The material parameters of the FE model were reconstructed by inverse modeling, minimizing the difference between the measured and the simulated deformation.

### Model Corneas

**Fig 1:**
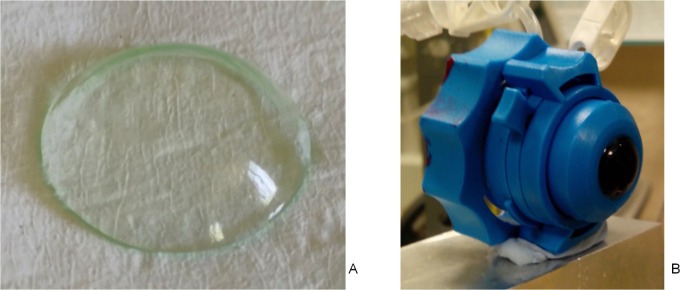
Model cornea (MC). A. Model cornea made out of hydrophilic contact lens material. B. Model cornea mounted in artificial eye chamber.

MCs of three soft, hydrophilic contact lens materials were studied (Fig 1). The MCs were lathed by contact lens manufacturer *mark’ennovy* (Madrid, Spain). The nominal physical properties of the materials are summarized in Table 1. The MCs were manufactured with three different, uniform thicknesses: 350 μm, 450 μm and 550 μm later on referred to as 1, 2 and 3, respectively. The anterior and posterior surfaces had spherical geometries with posterior radius of curvature of 8.60, and anterior radii of 8.95, 9.05 and 9.15 mm, respectively.

**Table 1:** Physical properties of Model Corneas (from manufacturer).

Short name	Gentle (G)	Saphir (S)	Quattro (Q)
**Brand name**	Gentle 80 Ori:gen	Saphir RX Spheric	Quattro Advantage 49
**Material composition**	Acrylic Co-Acrylamide Ter-Polymer	Silicone hydrogel	Co-polymer of HEMA & GMA
**ISO Hydrogel Name**	Filcon II 3	Filcon V 3	Filcon I 1
**Water Content**	79% ± 2	75% ± 1	49% ± 2
**Young’s Modulus [MPa]**	0.16	0.3	0.38
**Elongation at break [%]**	221	269	200

**Fig 2:**
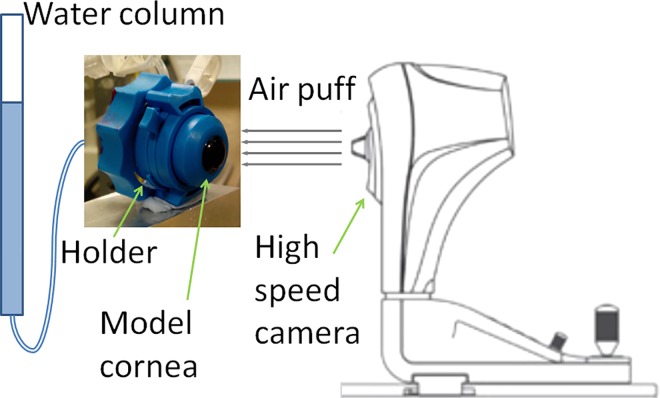
Schematic of the air puff test configuration.

The MCs were mounted (Fig 1B) in a plastic artificial eye chamber (Barron, Katena, Denville, NJ, USA), filled in with saline solution, and pressurized by a water column system to model the IOP (Fig 2). The pressure was set to 15 mm Hg in all cases. MCs were stained in fluorescein (0.1%) for 30 s before mounted in the holder. As MCs were made of hydrophilic materials, their hydration state is of importance to maintain mechanical properties, and therefore they were wetted by spraying water on them once every 60 s, while the internal surface was continuously in contact with the solution inside the artificial eye.

### Porcine corneas

Two porcine eyes from the same animal (age 6 months), obtained from a local slaughterhouse (Madrid, Spain) were tested. Measurements started within 4 hours post-mortem, and the eyes were kept on ice in a cooler box before the sample preparations. The corneas (P1 and P2) were excised and mounted in the artificial eye chamber; they were wetted by spraying water on them once every 60 s in order to avoid de-hydration. The eyes had a central corneal thickness of 724 μm for P1 and 625 μm for P2 (measured by Corvis ST).

After the air puff deformation imaging strips with a width of 3 mm were cut from the central part of the cornea along the horizontal meridian from white to white. The strips were measured by a uniaxial materials testing machine within 30 min after the air puff tests.

### Air puff deformation imaging

**Fig 3:**
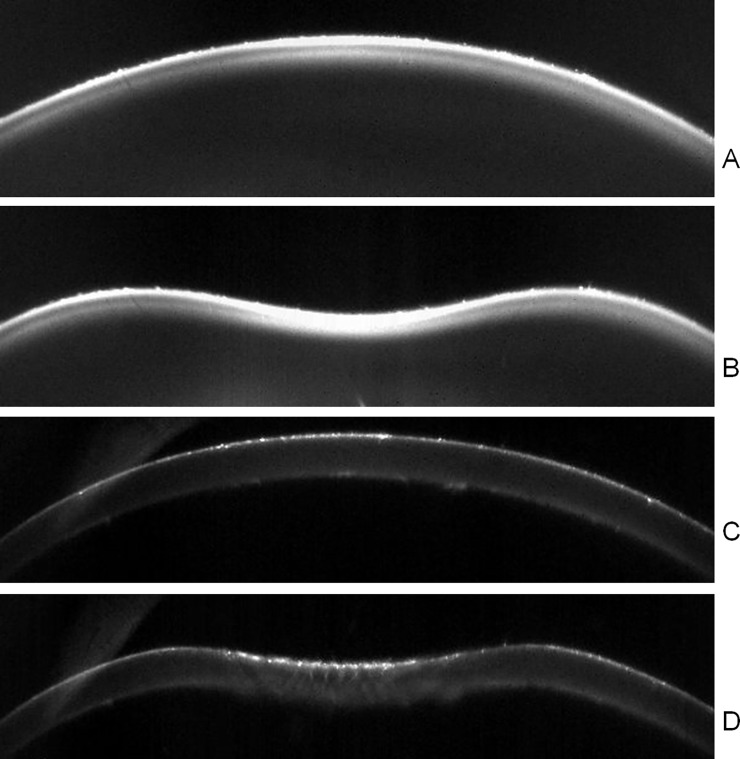
**Cross-sections of a MC (S2, A-B) and a porcine cornea (C-D).** A and C. Before the air puff deformation. B and D. At highest concavity

**Fig 4:**
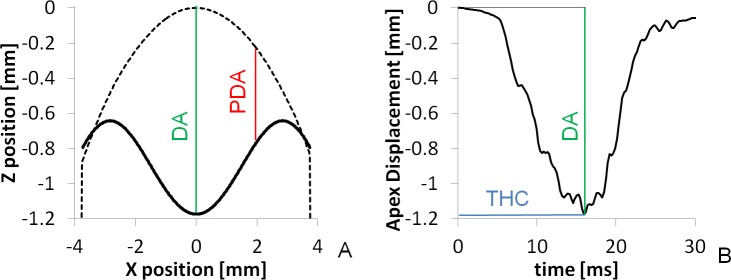
Spatial and temporal deformation profiles. A. Spatial deformation profile of Model Cornea Saphir 1 (Solid line). Dashed line shows the initial shape of the MC. B. Temporal deformation profile for the same MC. Deformation parameters are indicated: Central Deformation Amplitude (DA, in green), Peripheral Deformation Amplitude (PDA, in red), Time to Highest Concavity (THC, in blue).

The Corvis ST (Oculus, Germany) combines a controlled air pulse to deform the cornea with an ultra high speed Scheimpflug imaging camera to capture the deformation event. The system takes 140 horizontal cross-sectional corneal images during the ~30-ms deformation event (i.e., at a rate of about 4330 images/second) with a resolution of 640 x 480 pixels. The air puff test configuration is depicted in Fig 2. Typical images provided by the Corvis ST are shown in Fig 3. Fig 3A shows MC S2 before the air puff deformation, Fig 3B shows the same MC at the fully deformed state, generally denoted as highest concavity. Fig 3C and 3D show a Corvis ST image of a porcine cornea before and at highest deformation, respectively. Several parameters are obtained during the air puff deformation tests; these are summarized in Table 2, and illustrated in Fig 4.

**Table 2:** Air puff deformation parameters from Corvis ST.

Parameter	Definition
**Central Deformation Amplitude (DA)**	Displacement of the apex at highest concavity (HC)
**Peripheral Deformation Amplitude (PDA)**	Average Displacement at 2 mm from the apex on both sides at HC
**Central- Peripheral Deformation Ratio (CPR)**	DA/PDA
**Time to Highest Concavity (THC)**	Time from start until HC

The segmented corneal anterior surface cross-sectional profile at HC (commonly referred to as spatial deformation profile) and the temporal deformation profiles (the displacement history of the apex) were exported and analyzed. Fig 4 shows examples of the spatial (A) and temporal (B) deformation profiles for one of the MCs, and illustration of relevant deformation parameters.

### Uniaxial tensile tests

Uniaxial tensile tests on hydrogel corneas were carried out in the Biomaterials and Regenerative Engineering Laboratory of Universidad Politécnica de Madrid using an Instron 5543A (Instron Corp, Norwood, MA, USA) universal mechanical testing machine. Sample strips were cut out from the 550 μm thick MCs of all three materials (sample dimensions: 16 mm length x 3 mm width x 0.55 mm thickness). The device was equipped with an immersion chamber and temperature control. Tests were done in water immersion at 30°C, at a rate of 1 mm/s until rupture. The free length between the clamps before stretching was 12 mm.

Uniaxial tensile tests were performed on porcine corneas after the air puff tests using a UStretch biomaterials testing machine (Cellscale, Waterloo, ON, Canada) at the Visual Optics and Biophotonics Lab (Instituto de Optica, CSIC). The test were carried out in immersion of saline solution at a temperature of 25°C, at a rate of 0.1 mm/s up to stretch of 2 mm (with a maximum force of 4 N). The free length of the samples between the clamps before stretching was 8 mm.

### Inverse modeling

Material parameters were obtained by an inverse modeling process incorporating numerical simulations, programmed in a commercial FE software package (Ansys Workbench R15, Ansys Inc., Canonsburg, PA).

**Fig 5:**
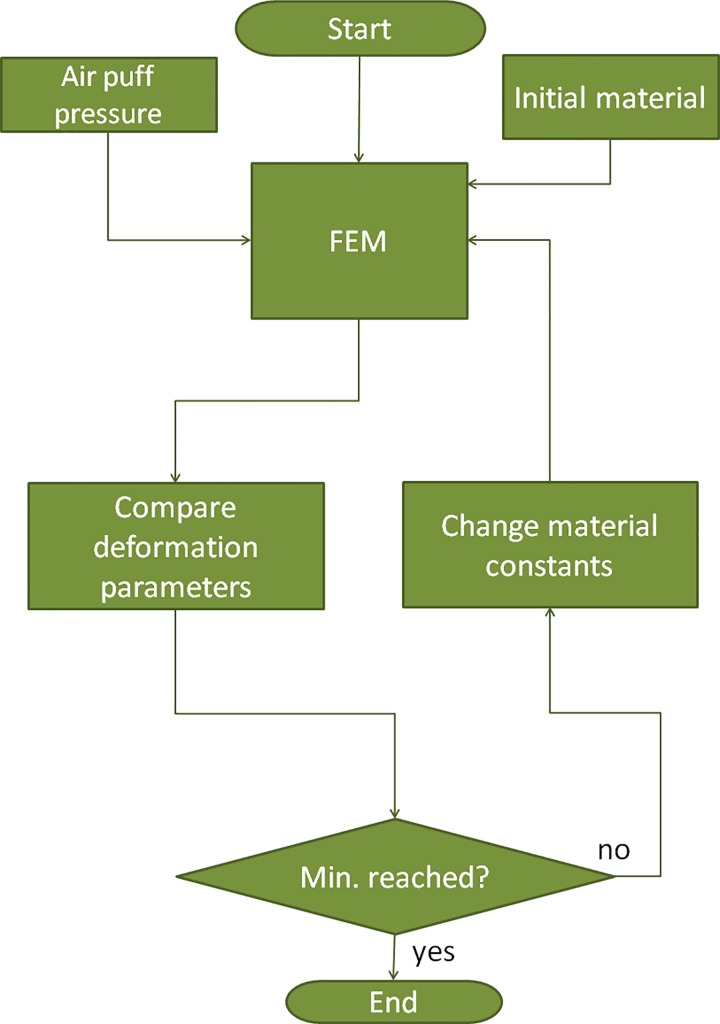
Flowchart of the inverse modeling process to retrieve the material parameters from air puff corneal deformation measurements.

Fig 5 shows the block diagram of the inverse modeling process. The pressure distribution of the air puff was determined in an earlier study [33]. First, a FE simulation is run using an initial set of material parameters. A merit function (Eq 1) containing deformation parameters DA and CPR was minimized in an iterative process, where DA_m_ and DA_sim_ are the measured and the simulated values of DA, respectively; CPR_m_ and CPR_sim_ are the measured and the simulated values of CPR, respectively. A built-in adaptive multiple-objective optimization was utilized that combines a Kriging response surface and a genetic algorithm [34]. The variables were two material parameters C_10_ and C_01_.

$$\Phi =|{DA}_{m}-{DA}_{sim}|+\frac{\left|{CRP}_{m}-{CRP}_{sim}\right|}{3}$$ (1)

**Fig 6:**
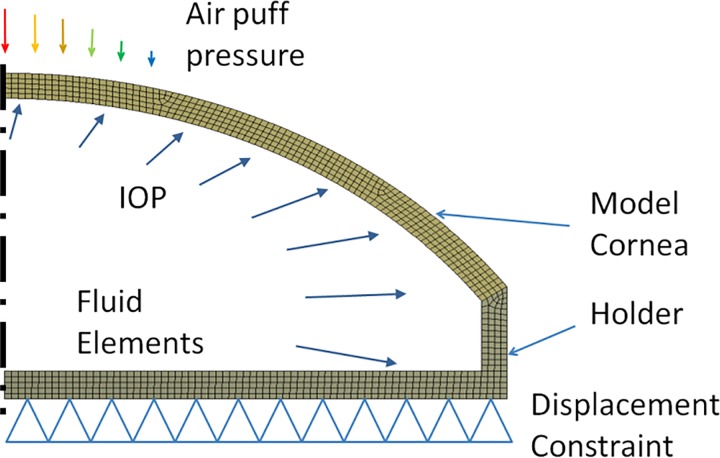
FE model of the model eye with the applied boundary conditions.

The FE model with the boundary conditions is depicted in Fig 6. Axial symmetry was assumed. The MC and the holder was modeled by 4-node elements (type: PLANE182). The liquid inside was modeled by incompressible fluid elements (type: HSFLD241). These fluid elements have an extra degree of freedom for pressure [34]. The IOP was applied as a pressure load on these elements. The MC was modeled by a hyperelastic Mooney-Rivlin material model, with two parameters: C_10_ and C_01_. The strain energy density function *W* for an incompressible Mooney–Rivlin material is:

$$W=C_{10}(I_{1}-3)+C_{01}(I_{2}-3)$$ (2)

where I_1_ and I_2_ are the first and the second invariant of the left Cauchy–Green deformation tensor. The holder was considered as a linear isotropic elastic body, with a Young’s modulus of E = 1500 MPa.

### Comparison with extensiometry data

In order to validate the inherent material properties obtained by the above method, the uniaxial tensile tests were modeled by means of FE. The material parameters from the inverse modeling process were incorporated in this model, and the stress-strain relation was plotted and compared to that of the experimental measurement. Statistical differences between methods were estimated by an ANOVA test.

## Results

### Experimental deformation parameters

**Fig 7:**
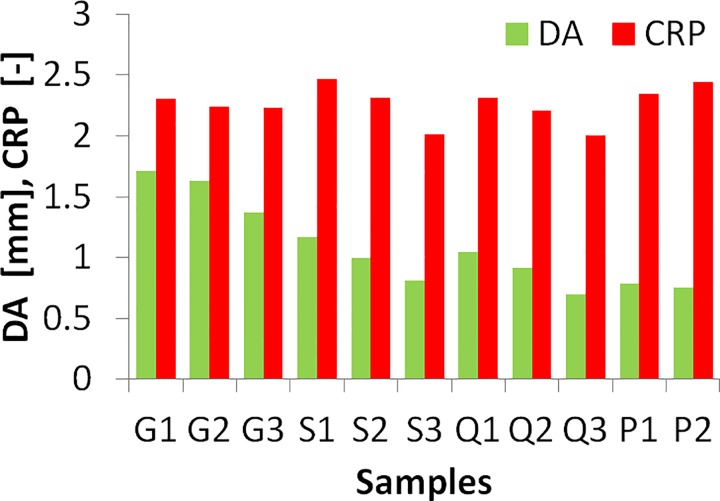
Experimental Deformation amplitude (DA) and Central-Peripheral Deformation Ration (CPR) from Corvis ST air puff tests, for all model corneas and the two porcine corneas.

Fig 7 shows the experimental DA and CPR results measured by the Corvis ST, at a constant IOP of 15 mm Hg. As expected, the thicker the MC the smaller the deformation (DA) in all materials. CPR also decreases with increasing thickness, to a lesser extent for the softest material (G). DA in porcine corneas was of the same order of magnitude than the thickest samples of the S and Q materials and CPR was closest to G and S thinnest samples. There were slight differences (4.5% and 3.9% for DA and CPR) between the two porcine eyes (even if thickness was similar).

### Reconstructed mechanical properties

**Fig 8:**
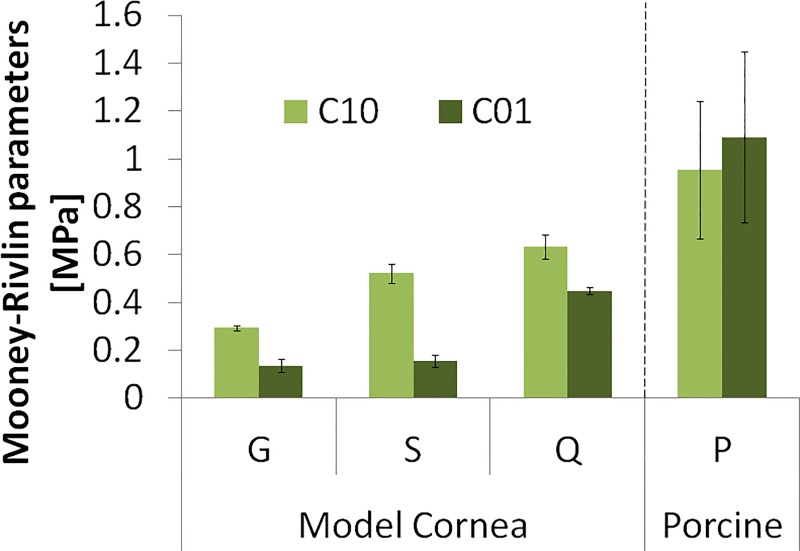
Inherent material properties C_10_ and C_01_ for model corneas of three different materials (G, S, Q) and porcine corneas (P). Error bars stand for the standard deviation of estimates from corneas of different thicknesses in the model corneas, and for the standard deviation across corneal samples.

The reconstructed parameters C_10_ and C_01_ of the hyperelastic Mooney-Rivlin material model (Eq 2) are shown in Fig 8. As expected Q is the hardest and G is the softest polymer material. The material properties reconstructed independently on corneas with the same material and different thicknesses are very consistent, with a variability of less than 7% for C_10_ and 13% for C_01_ (error bars in Fig 8), averaged across materials. Porcine corneas are harder than the MCs, and material properties showed a larger standard deviation across eyes (even from the same animal), 30% for C_10_ and 33% for C_01_(error bars in Fig 8).

### Experimental and simulated deformation profiles

**Fig 9:**
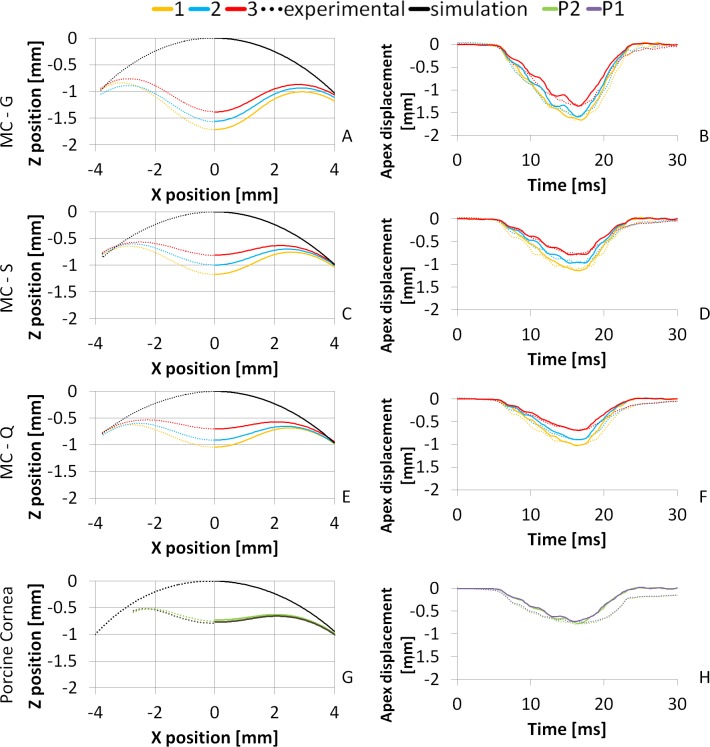
Spatial (left) and temporal (right) deformation profiles for three model corneas and three thicknesses; and the porcine corneas. A, B: Material G; C, D: Material S; E, F: Material Q; G, H: Porcine (P1 and P2). Black lines represent the initial profile; yellow, blue and red represent the 1, 2 and 3 MC thicknesses (350, 450, 550 μm), respectively. Green and Purple lines represent the two porcine corneas. Dotted lines stand for the measured profile and solid lines stand for the simulated profile using the reconstructed material parameters.

The reconstructed material parameters were used to simulate the corresponding air puff deformation profiles. Fig 9 compares the experimental (dotted lines) and simulated (solid lines) spatial and deformation profiles of model corneas, for all three thicknesses (1, yellow; 2 blue; 3, red) and all three materials (G, A-B; S, C-D; Q, E-F), as well as of porcine corneas (P, G-H). The average RMS difference between experimental and simulated profiles is 0.04 mm (spatial profiles) and 0.1 mm (temporal profiles) in model polymer corneas and 0.08 mm (spatial profiles) and 0.14 mm (temporal profiles) in porcine corneas.

### Experimental and simulated uniaxial tensile tests

The inherent material properties obtained from the inverse modeling process were introduced in the FE model of the uniaxial tensile test, simulating strips of the same dimensions as used in the experimental tensile tests.

**Fig 10:**
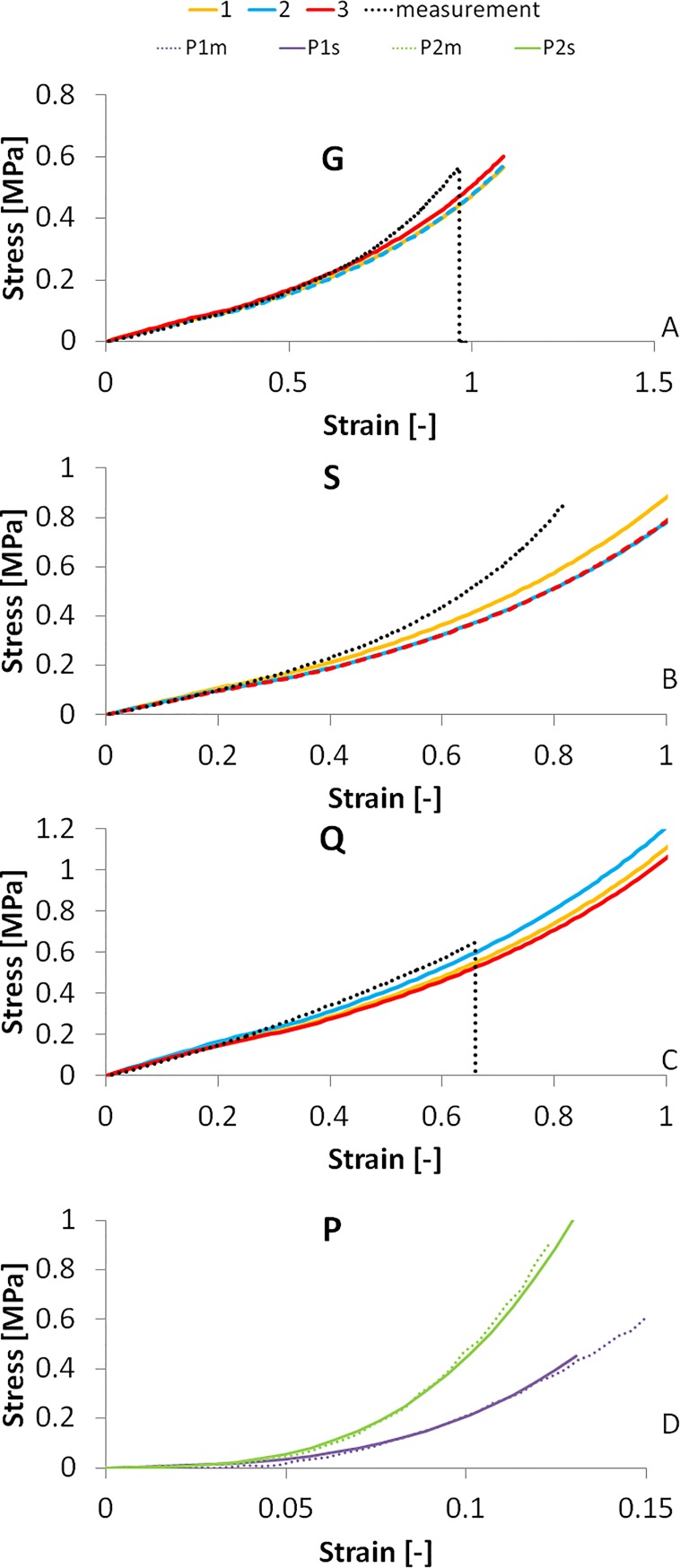
Stress-strain curves of the MC materials and porcine corneas by the inverse modeling from air-puff measurements and from uniaxial extensiometry measurements. A: Material G; B: Material S; C: Material Q; D: Porcine corneas.

Fig 10 shows the stress-strain curves of the materials obtained by the simulations of the model corneas and the experimental stress-stain curves, for all three materials measurement. The stress-strain curves were simulated using the material parameters reconstructed from MC of different thicknesses (each represented by a color in Fig 10A–10C) and from two porcine corneas (Fig 10D). In polymer model corneas, the average point-to-point correspondence of the experimental and simulated stress-strain curves in the tested strain ranges (0–0.4) is 95.15% for G, 93.52% for S and 95.5% for Q. Note that in the air puff deformation the maximum equivalent strain is less than 0.3, which corresponds to approximately 3 mm of stretching.

**Fig 11:**
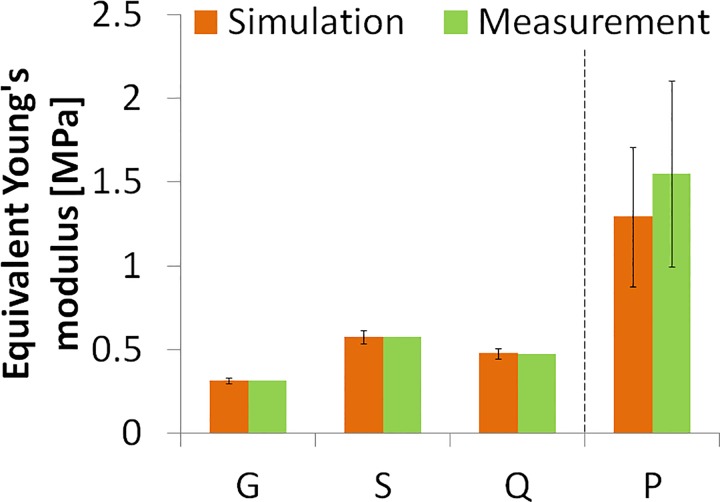
Equivalent Young's moduli from stress-strain measured and simulated stress-strain curves. Simulated curves use material properties obtained from inverse modeling from air puff data at different thicknesses. Error bars in the simulation data represent the standard deviation across the parameter values from air puff deformation based estimates on corneal samples of different thicknesses. Error bar in measurement data of the porcine cornea (P) represent the standard deviation across samples (n = 2).

For a better comparison with other measurements, we calculated the equivalent Young’s moduli (estimated as the average slope of the stress-strain curves in the 0–0.1 strain range) in both the measured and simulated stress-strain curves, as shown in Fig 11. There is an excellent agreement between simulated and measured Young’s modulus (99.23% correspondence), and less than 7% variability of data obtained from different thickness model polymer corneas in the air puff experiment (error bars in Fig 11). ANOVA test did not find statistically significant differences in the Young’s modulus obtained from either method (p = 0.98). Fig 11 also shows the equivalent Young’s modulus calculated for the porcine corneas (last column, P). The intersubject variability of experimental extensiometry data in porcine corneas is higher than in model corneas, as also found in the estimates from the reconstructed material properties using air-puff deformation. The reconstructed equivalent Young’s modulus from air puff (0.99 MPa for P1 and 1.59 MPa for P2) were lower than those measured by uniaxial extensiometry, by 13% and 18%, for P1 and P2 respectively. This difference between methods is significantly lower than the intersubject difference and the ANOVA test did not find statistically significant differences in the equivalent Young’s modulus values obtained from either method (p = 0.65).

## Discussion

Measuring the biomechanical corneal properties of the cornea has great value for diagnostics, for improving the predictability of treatments and for post-treatment monitoring. Air puff corneal deformation imaging has the potential for in vivo evaluation of mechanical properties, yet most studies to date using this technique report corneal deformation parameters, but not inherent corneal mechanical properties. In this study, we report for the first time to our knowledge the accuracy of air puff corneal deformation imaging in combination with inverse modeling to retrieve mechanical parameters. We showed a high correspondence between the Young’s modulus and hyperelastic constants obtained from our technique and standard uniaxial tensile tests.

The method is demonstrated on model corneas made out of contact lens materials. In many regards, these model corneas represent typical behavior of real corneas. We selected the thickness of the MCs in ranges typically found in human corneas (around 500 μm in normal corneas and below 400 μm in keratoconic or post-LASIK corneas). The air puff deformation parameters were similar to those of the human corneas: DA varied between 0.7 mm and 1.6 mm in our MCs, while Valbon et al. measured DA between 0.9 and 1.26 mm in healthy human corneas using Corvis ST air puff deformation [35]. Some mechanical properties are also comparable between MCs and the real corneas. Both show non-linear characteristics, and the stress-strain curves have similar shapes. A typical stress-strain curve of the cornea is convex, steepening progressively at higher strains [36], explained by the fact that the collagen fibers in the corneal stroma bend easily, but they are relatively stiff when subjected to tensile load. As the cornea is stretched, an increasing number of fibers get oriented and aligned with the load, resulting in higher slopes (stiffening) in the stress-strain curves for increased strain. MC materials show similar effect at higher strains, because (similarly to other amorphous polymers) are composed of long, randomly oriented molecule chains, which get oriented by stretching the material. Although the stress-strain curves of the MCs are very similar to those of the corneas, their tensile strength is nevertheless significantly lower.

Temporal parameters of the air puff deformation of the MCs also differ from those of human or animal corneas. The average time to highest concavity (HC) was 16.2 ms (± 0.24), while 18.38 ms has been reported for human corneas [35] and 17 ms in rabbits [8]. The shape of the temporal profiles is also different, being very symmetrical and steeper than real corneas in MCs (Fig 9 right), following closely the air puff pressure profile over time. These differences suggest that MCs exhibit significantly less time-dependent mechanical behavior, i.e. lower viscoelasticity than human or animal corneas. Considering this, viscosity was not taken into account in the material models of the mechanical simulations, although real corneas are better described by a viscoelastic model. Also, the model corneas are isotropic unlike real corneas in which the different meridional alignment and density of the collagen fibers result in anisotropic behavior. As only cross-sectional corneal meridians are imaged and the model is two-dimensional this aspect is of secondary importance in our setting.

We also applied the air-puff corneal deformation and inverse modeling method to reconstruct inherent properties of porcine corneas, and compared them to standard uniaxial extensiometry performed on the same corneas. We found equivalent Young’s modulus of 0.99–1.59 MPa (from air-puff) and 1.15–1.93 MPa (from uniaxial extensiometry). Both methods captured the inter-eye differences of the measured samples. On the other hand the reconstructed values are in agreement with previous reports in the literature on porcine corneas: 0.8–2.6 MPa from Wollensak et al. using uniaxial extensiometry [37]; 0.6–3.9 MPa from Kling et al, using eye’s inflation [15]. The differences between the measured and simulated results may come from the assumptions of the material models. In the model viscoelasticity and anisotrophy were not considered although real corneas do show time- [38] and orientation-dependent [39] mechanical behavior. The higher variability in real corneas may in fact arise from differences in the measured corneal meridian (between eyes, and even between the imaged cross-section in the air-puff corneal imaging and the orientation of the excised strip for corneal extensiometry). Although the horizontal meridian was targeted in all cases, the corneal excision may compromise proper identification of the actual corneal orientation. Previous literature has shown significant meridional differences in the corneal collagen distribution in the porcine cornea [40] similarly to human corneas [41, 42], resulting in meridional variations in elasticity [43].

In general, the material model of choice seems appropriate to describe the polymer corneas, and to a great extent to describe the porcine corneas. Although the results suggest that an anistropic model would be a better choice to describe full corneas, at present the method is limited by the 2-D nature of the techniques of use (the air-puff corneal deformation imaging only captures images along the horizontal meridian, and uniaxial extensiometry measures only properties along one direction). In previous publications we have used viscoelastic models, which were preferred in particular to assess mechanical changes in cross-linked corneas.

The reconstructed material properties are in good agreement with the tensile test results, especially in the lower strain region. The deviations found at higher strain levels, which are beyond the maximum equivalent strain at highest deformation (between 0.1 and 0.3, corresponding approximately to 3–5 mm of stretching in uniaxial tension) in the air-puff test. The reconstructed material parameters in the hydrogel polymer corneal models from the same material but different thickness prove the robustness of the proposed method. On the other hand, the validation in porcine corneas proves that the method can be reliably applied in real corneas, without the simplification of the models. The exceptionally good fit between the equivalent Young’s modulus values proves that the proposed method can reconstruct the material properties with high accuracy. Air puff corneal deformation imaging and inverse modeling can therefore be used reliably to obtain mechanical properties of materials exhibiting overall similar deformation properties with those of the cornea as well as on frequently used animal cornea model, and is a promising quantification tool in the clinic.

## Conclusions

Air puff corneal deformation imaging has been combined with inverse finite element modeling technique to retrieve the inherent mechanical properties of hydrogel polymer model corneas and porcine corneas. The algorithm provided similar inherent polymer material parameters for three different thicknesses in independent simulations. The method has been validated with three different materials and real corneas. Simulations using the retrieved material properties accurately reproduced experimental air puff temporal and spatial corneal deformation profiles and uniaxial tensile stress-strain curves.

## Supporting Information

S1 Dataset Dataset containing temporal and spatial profiles, and stress-strain relations of the studied model and porcine corneas. (XLSX)
